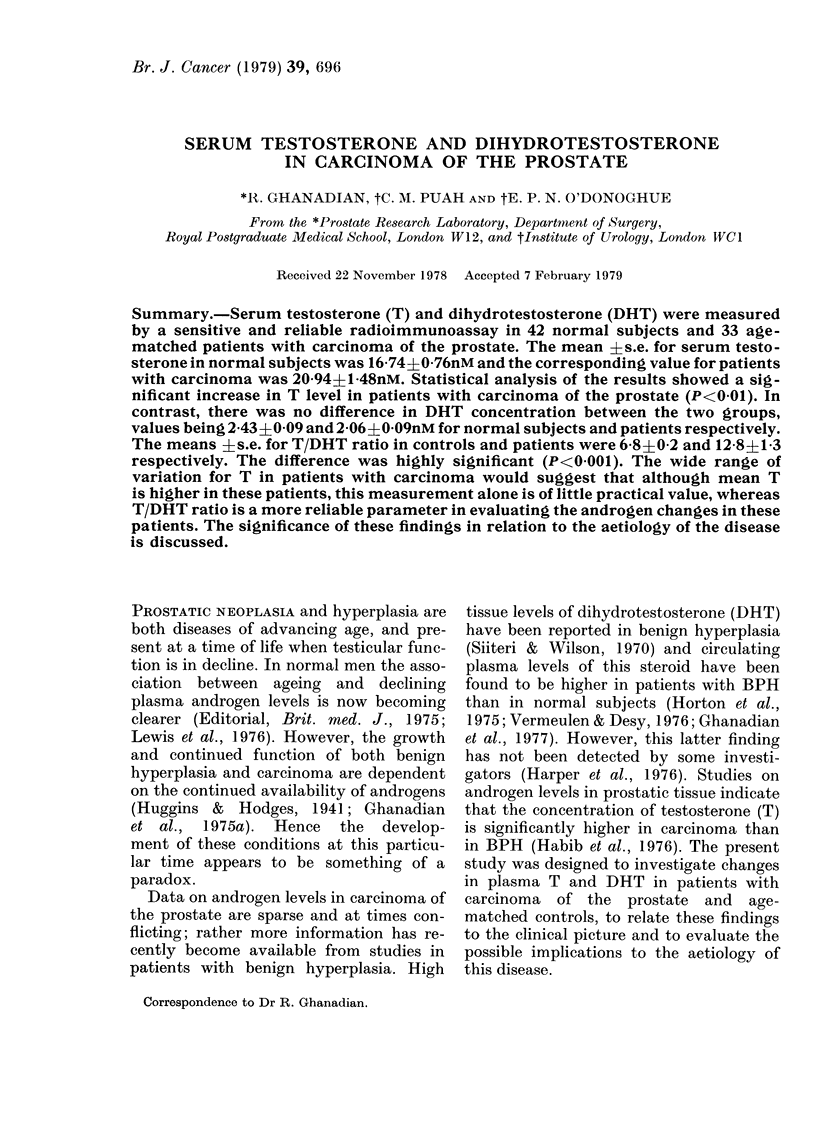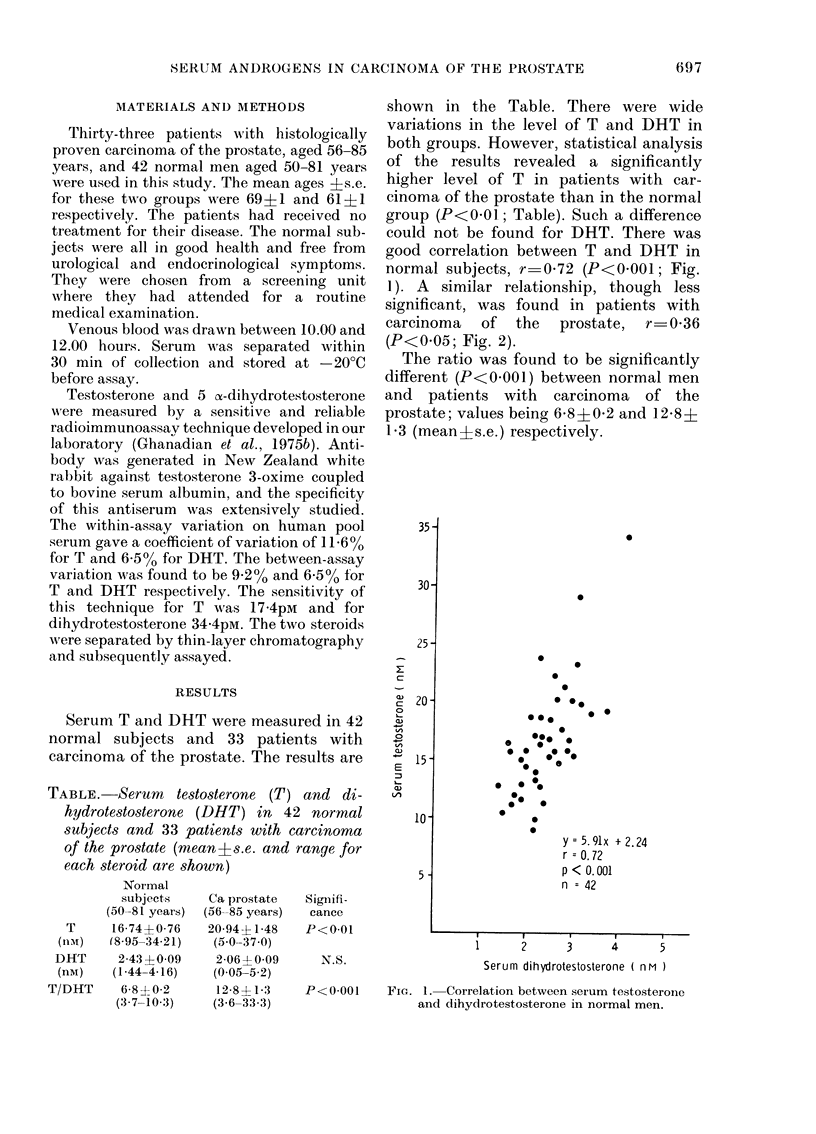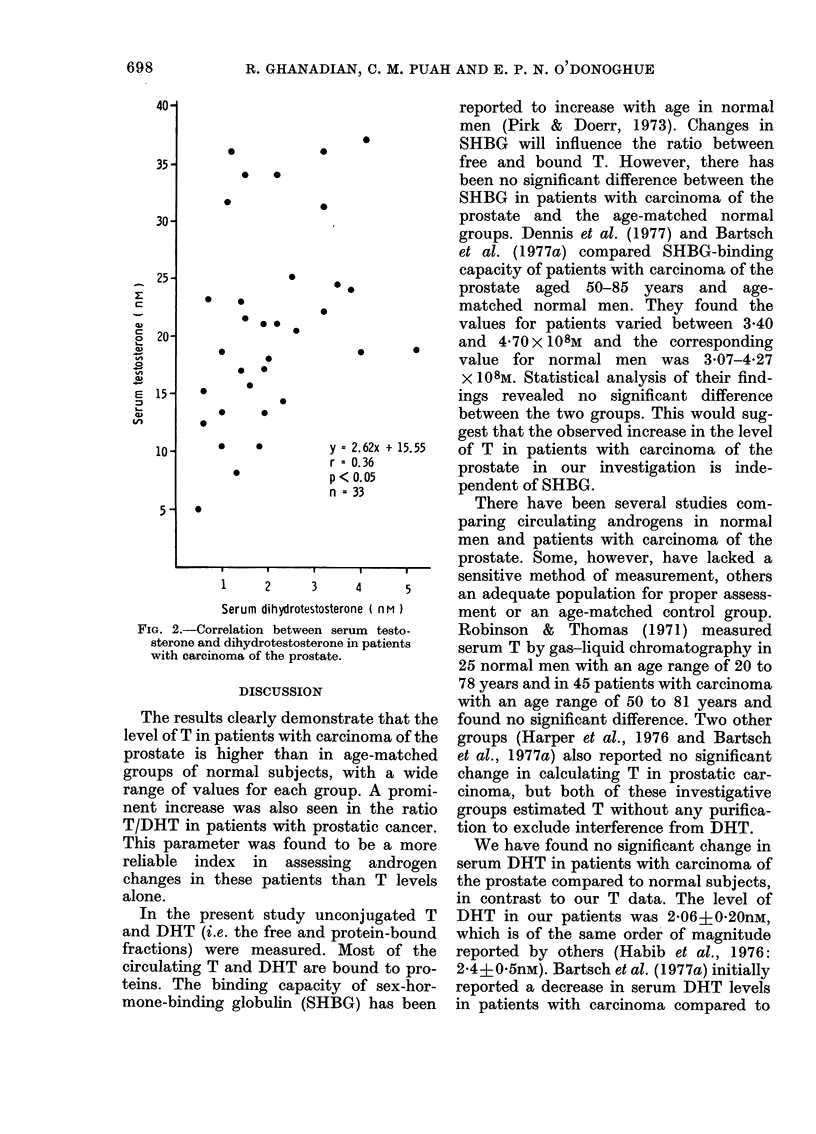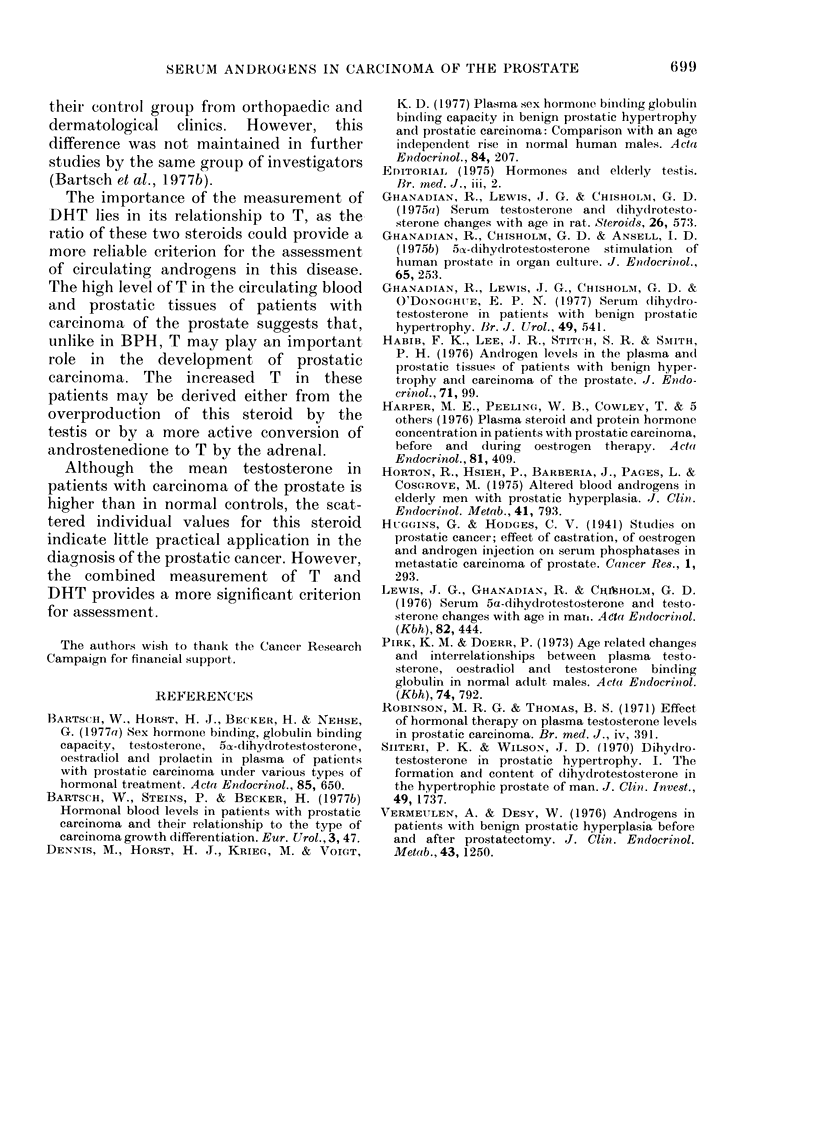# Serum testosterone and dihydrotestosterone in carcinoma of the prostate.

**DOI:** 10.1038/bjc.1979.122

**Published:** 1979-06

**Authors:** R. Ghanadian, C. M. Puah, E. P. O'Donoghue

## Abstract

Serum testosterone (T) and dihydrotestosterone (DHT) were measured by a sensitive and reliable radioimmunoassay in 42 normal subjects and 33 age-matched patients with carcinoma of the prostate. The mean +/- s.e. for serum testosterone in normal subjects was 16.74 +/- 0.76nM and the corresponding value for patients with carcinoma was 20.94 +/- 1.48nM. Statistical analysis of the results showed a significant increase in T level in patients with carcinoma of the prostate (P less than 0.01). In contrast, there was no difference in DHT concentration between the two groups, values being 2.43 +/- 0.09 and 2.06 +/- 0.09nM for normal subjects and patients respectively. The means +/- s.e. for T/DHT ratio in controls and patients were 6.8 +/- 0.2 and 12.8 +/- 1.3 respectively. The difference was highly significant (P less than 0.001). The wide range of variation for T in patients with carcinoma would suggest that although mean T is higher in these patients, this measurement alone is of little practical value, whereas T/DHT ratio is a more reliable parameter in evaluating the androgen changes in these patients. The significance of these findings in relation to the aetiology of the disease is discussed.


*Br. J. Cancer* (1979) **39**, 696

### SERUM TESTOSTERONE AND DIHYDROTESTOSTERONE IN CARCINOMA OF THE PROSTATE

*R. GHANADIAN, †C. M. PUAH AND †E. P. N. O'DONOGHUE

*From the *Prostate Research Laboratory, Department of Surgery, Royal Postgraduate Medical School, London W12, and †Institute of Urology, London WC1*

Received 22 November 1978 Accepted 7 February 1979

**Summary.—Serum testosterone (T) and dihydrotestosterone (DHT) were measured by a sensitive and reliable radioimmunoassay in 42 normal subjects and 33 age-matched patients with carcinoma of the prostate. The mean ±s.e. for serum testosterone in normal subjects was 16·74±0·76nM and the corresponding value for patients with carcinoma was 20·94±1·48nM. Statistical analysis of the results showed a significant increase in T level in patients with carcinoma of the prostate ($P<0·01$). In contrast, there was no difference in DHT concentration between the two groups, values being 2·43±0·09 and 2·06±0·09nM for normal subjects and patients respectively. The means ±s.e. for T/DHT ratio in controls and patients were 6·8±0·2 and 12·8±1·3 respectively. The difference was highly significant ($P<0·001$). The wide range of variation for T in patients with carcinoma would suggest that although mean T is higher in these patients, this measurement alone is of little practical value, whereas T/DHT ratio is a more reliable parameter in evaluating the androgen changes in these patients. The significance of these findings in relation to the aetiology of the disease is discussed.**

PROSTATIC NEOPLASIA and hyperplasia are both diseases of advancing age, and present at a time of life when testicular function is in decline. In normal men the association between ageing and declining plasma androgen levels is now becoming clearer (Editorial, *Brit. med. J.*, 1975; Lewis *et al.*, 1976). However, the growth and continued function of both benign hyperplasia and carcinoma are dependent on the continued availability of androgens (Huggins & Hodges, 1941; Ghanadian *et al.*, 1975*a*). Hence the development of these conditions at this particular time appears to be something of a paradox.

Data on androgen levels in carcinoma of the prostate are sparse and at times conflicting; rather more information has recently become available from studies in patients with benign hyperplasia. High tissue levels of dihydrotestosterone (DHT) have been reported in benign hyperplasia (Siiteri & Wilson, 1970) and circulating plasma levels of this steroid have been found to be higher in patients with BPH than in normal subjects (Horton *et al.*, 1975; Vermeulen & Desy, 1976; Ghanadian *et al.*, 1977). However, this latter finding has not been detected by some investigators (Harper *et al.*, 1976). Studies on androgen levels in prostatic tissue indicate that the concentration of testosterone (T) is significantly higher in carcinoma than in BPH (Habib *et al.*, 1976). The present study was designed to investigate changes in plasma T and DHT in patients with carcinoma of the prostate and age-matched controls, to relate these findings to the clinical picture and to evaluate the possible implications to the aetiology of this disease.

Correspondence to Dr R. Ghanadian.

### MATERIALS AND METHODS

Thirty-three patients with histologically proven carcinoma of the prostate, aged 56–85 years, and 42 normal men aged 50–81 years were used in this study. The mean ages ±s.e. for these two groups were 69±1 and 61±1 respectively. The patients had received no treatment for their disease. The normal subjects were all in good health and free from urological and endocrinological symptoms. They were chosen from a screening unit where they had attended for a routine medical examination.

Venous blood was drawn between 10.00 and 12.00 hours. Serum was separated within 30 min of collection and stored at −20°C before assay.

Testosterone and 5 α-dihydrotestosterone were measured by a sensitive and reliable radioimmunoassay technique developed in our laboratory (Ghanadian *et al.*, 1975*b*). Antibody was generated in New Zealand white rabbit against testosterone 3-oxime coupled to bovine serum albumin, and the specificity of this antiserum was extensively studied. The within-assay variation on human pool serum gave a coefficient of variation of 11·6% for T and 6·5% for DHT. The between-assay variation was found to be 9·2% and 6·5% for T and DHT respectively. The sensitivity of this technique for T was 17·4pM and for dihydrotestosterone 34·4pM. The two steroids were separated by thin-layer chromatography and subsequently assayed.

### RESULTS

Serum T and DHT were measured in 42 normal subjects and 33 patients with carcinoma of the prostate. The results are shown in the Table. There were wide variations in the level of T and DHT in both groups. However, statistical analysis of the results revealed a significantly higher level of T in patients with carcinoma of the prostate than in the normal group ($P<0{\cdot}01$; Table). Such a difference could not be found for DHT. There was good correlation between T and DHT in normal subjects, $r=0{\cdot}72$ ($P<0{\cdot}001$; Fig. 1). A similar relationship, though less significant, was found in patients with carcinoma of the prostate, $r=0{\cdot}36$ ($P<0{\cdot}05$; Fig. 2).

The ratio was found to be significantly different ($P<0{\cdot}001$) between normal men and patients with carcinoma of the prostate; values being 6·8±0·2 and 12·8±1·3 (mean±s.e.) respectively.

TABLE.—*Serum testosterone (T) and dihydrotestosterone (DHT) in 42 normal subjects and 33 patients with carcinoma of the prostate (mean±s.e. and range for each steroid are shown)*

| | Normal subjects (50–81 years) | Ca prostate (56–85 years) | Significance |
|---|---|---|---|
| T (nM) | 16·74±0·76 (8·95–34·21) | 20·94±1·48 (5·0–37·0) | $P<0{\cdot}01$ |
| DHT (nM) | 2·43±0·09 (1·44–4·16) | 2·06±0·09 (0·05–5·2) | N.S. |
| T/DHT | 6·8±0·2 (3·7–10·3) | 12·8±1·3 (3·6–33·3) | $P<0{\cdot}001$ |

FIG. 1.—Correlation between serum testosterone and dihydrotestosterone in normal men.

FIG. 2.—Correlation between serum testosterone and dihydrotestosterone in patients with carcinoma of the prostate.

### DISCUSSION

The results clearly demonstrate that the level of T in patients with carcinoma of the prostate is higher than in age-matched groups of normal subjects, with a wide range of values for each group. A prominent increase was also seen in the ratio T/DHT in patients with prostatic cancer. This parameter was found to be a more reliable index in assessing androgen changes in these patients than T levels alone.

In the present study unconjugated T and DHT (*i.e.* the free and protein-bound fractions) were measured. Most of the circulating T and DHT are bound to proteins. The binding capacity of sex-hormone-binding globulin (SHBG) has been reported to increase with age in normal men (Pirk & Doerr, 1973). Changes in SHBG will influence the ratio between free and bound T. However, there has been no significant difference between the SHBG in patients with carcinoma of the prostate and the age-matched normal groups. Dennis *et al.* (1977) and Bartsch *et al.* (1977*a*) compared SHBG-binding capacity of patients with carcinoma of the prostate aged 50–85 years and age-matched normal men. They found the values for patients varied between 3·40 and $4{\cdot}70 \times 10^{8}$M and the corresponding value for normal men was 3·07–4·27 $\times 10^{8}$M. Statistical analysis of their findings revealed no significant difference between the two groups. This would suggest that the observed increase in the level of T in patients with carcinoma of the prostate in our investigation is independent of SHBG.

There have been several studies comparing circulating androgens in normal men and patients with carcinoma of the prostate. Some, however, have lacked a sensitive method of measurement, others an adequate population for proper assessment or an age-matched control group. Robinson & Thomas (1971) measured serum T by gas–liquid chromatography in 25 normal men with an age range of 20 to 78 years and in 45 patients with carcinoma with an age range of 50 to 81 years and found no significant difference. Two other groups (Harper *et al.*, 1976 and Bartsch *et al.*, 1977*a*) also reported no significant change in calculating T in prostatic carcinoma, but both of these investigative groups estimated T without any purification to exclude interference from DHT.

We have found no significant change in serum DHT in patients with carcinoma of the prostate compared to normal subjects, in contrast to our T data. The level of DHT in our patients was $2{\cdot}06 \pm 0{\cdot}20$nM, which is of the same order of magnitude reported by others (Habib *et al.*, 1976: $2{\cdot}4 \pm 0{\cdot}5$nM). Bartsch *et al.* (1977*a*) initially reported a decrease in serum DHT levels in patients with carcinoma compared to their control group from orthopaedic and dermatological clinics. However, this difference was not maintained in further studies by the same group of investigators (Bartsch *et al.*, 1977*b*).

The importance of the measurement of DHT lies in its relationship to T, as the ratio of these two steroids could provide a more reliable criterion for the assessment of circulating androgens in this disease. The high level of T in the circulating blood and prostatic tissues of patients with carcinoma of the prostate suggests that, unlike in BPH, T may play an important role in the development of prostatic carcinoma. The increased T in these patients may be derived either from the overproduction of this steroid by the testis or by a more active conversion of androstenedione to T by the adrenal.

Although the mean testosterone in patients with carcinoma of the prostate is higher than in normal controls, the scattered individual values for this steroid indicate little practical application in the diagnosis of the prostatic cancer. However, the combined measurement of T and DHT provides a more significant criterion for assessment.

The authors wish to thank the Cancer Research Campaign for financial support.

### REFERENCES

BARTSCH, W., HORST, H. J., BECKER, H. & NEHSE, G. (1977*a*) Sex hormone binding, globulin binding capacity, testosterone, 5α-dihydrotestosterone, oestradiol and prolactin in plasma of patients with prostatic carcinoma under various types of hormonal treatment. *Acta Endocrinol.*, **85**, 650.

BARTSCH, W., STEINS, P. & BECKER, H. (1977*b*) Hormonal blood levels in patients with prostatic carcinoma and their relationship to the type of carcinoma growth differentiation. *Eur. Urol.*, **3**, 47.

DENNIS, M., HORST, H. J., KRIEG, M. & VOIGT, K. D. (1977) Plasma sex hormone binding globulin binding capacity in benign prostatic hypertrophy and prostatic carcinoma: Comparison with an age independent rise in normal human males. *Acta Endocrinol.*, **84**, 207.

EDITORIAL (1975) Hormones and elderly testis. *Br. med. J.*, iii, 2.

GHANADIAN, R., LEWIS, J. G. & CHISHOLM, G. D. (1975*a*) Serum testosterone and dihydrotestosterone changes with age in rat. *Steroids*, **26**, 573.

GHANADIAN, R., CHISHOLM, G. D. & ANSELL, I. D. (1975*b*) 5α-dihydrotestosterone stimulation of human prostate in organ culture. *J. Endocrinol.*, **65**, 253.

GHANADIAN, R., LEWIS, J. G., CHISHOLM, G. D. & O'DONOGHUE, E. P. N. (1977) Serum dihydrotestosterone in patients with benign prostatic hypertrophy. *Br. J. Urol.*, **49**, 541.

HABIB, F. K., LEE, J. R., STITCH, S. R. & SMITH, P. H. (1976) Androgen levels in the plasma and prostatic tissues of patients with benign hypertrophy and carcinoma of the prostate. *J. Endocrinol.*, **71**, 99.

HARPER, M. E., PEELING, W. B., COWLEY, T. & 5 others (1976) Plasma steroid and protein hormone concentration in patients with prostatic carcinoma, before and during oestrogen therapy. *Acta Endocrinol.*, **81**, 409.

HORTON, R., HSIEH, P., BARBERIA, J., PAGES, L. & COSGROVE, M. (1975) Altered blood androgens in elderly men with prostatic hyperplasia. *J. Clin. Endocrinol. Metab.*, **41**, 793.

HUGGINS, G. & HODGES, C. V. (1941) Studies on prostatic cancer; effect of castration, of oestrogen and androgen injection on serum phosphatases in metastatic carcinoma of prostate. *Cancer Res.*, **1**, 293.

LEWIS, J. G., GHANADIAN, R. & CHISHOLM, G. D. (1976) Serum 5α-dihydrotestosterone and testosterone changes with age in man. *Acta Endocrinol. (Kbh)*, **82**, 444.

PIRK, K. M. & DOERR, P. (1973) Age related changes and interrelationships between plasma testosterone, oestradiol and testosterone binding globulin in normal adult males. *Acta Endocrinol. (Kbh)*, **74**, 792.

ROBINSON, M. R. G. & THOMAS, B. S. (1971) Effect of hormonal therapy on plasma testosterone levels in prostatic carcinoma. *Br. med. J.*, iv, 391.

SIITERI, P. K. & WILSON, J. D. (1970) Dihydrotestosterone in prostatic hypertrophy. I. The formation and content of dihydrotestosterone in the hypertrophic prostate of man. *J. Clin. Invest.*, **49**, 1737.

VERMEULEN, A. & DESY, W. (1976) Androgens in patients with benign prostatic hyperplasia before and after prostatectomy. *J. Clin. Endocrinol. Metab.*, **43**, 1250.